# Subjective and neural reactivity during savoring and rumination

**DOI:** 10.3758/s13415-023-01123-2

**Published:** 2023-09-19

**Authors:** Benjamin O. Brandeis, Greg J. Siegle, Peter Franzen, Adriane Soehner, Brant Hasler, Dana McMakin, Kym Young, Daniel J. Buysse

**Affiliations:** 1 Dickinson College, Carlisle, PA USA; 2 University of Pittsburgh, School of Medicine, WPH, 3811 O’Hara St., Pittsburgh, PA 15213 USA; 3 Florida International University, Miami, FL USA

**Keywords:** Affect, Savor, Ruminate, fMRI

## Abstract

Repetitive thinking about negative emotions or events is strongly associated with worse mental health, whereas repetitive positive thought is generally believed to be beneficial. This observation is at odds with the idea that all forms of repetitive thinking share underlying neural mechanisms. To resolve this apparent discrepancy, the present study examined relationships between subjective affect and neural mechanisms during periods of sustained processing of positive (savoring) and negative (rumination) emotion. We also examined potential common moderators of savoring and rumination including memory specificity and sleep quality. Results indicated that individuals who experience high positive affect during savoring also are likely to experience more intense negative affect during rumination. fMRI-derived brain activity revealed common mechanisms of rumination and savoring. Memory specificity had common effects on neural correlates of rumination and savoring; sleep quality was not associated with mechanisms of savoring or rumination. These results suggest that repetitive engagement with positive and negative affect is similar both subjectively and mechanistically. Clinical interventions for rumination may benefit from capitalizing on preserved capacity for savoring.

**Supplementary Information:**

The online version contains supplementary material available at 10.3758/s13415-023-01123-2.

## Introduction

Sustained emotional information processing has been extensively studied, with relatively separate literatures for sustained/repetitive processing of positive information (e.g., savoring, (Bryant, 2021; Reis et al., 2010; Straszewski and Siegel, 2021)) and negative information (e.g., rumination (Edge et al., 2021)). Sustained processing of positive information is generally thought to be adaptive and beneficial; it decreases psychiatric symptomatology (Bryant et al., 2021) and protects against development of depression (Boelen and Lenferink, 2020; Yang and Li, 2020). In contrast, sustained processing of negative information is thought to be maladaptive; it increases the duration and severity of psychiatric symptoms (Lavoy, 2010; Papageorgiou and Wells, 2004) as well as vulnerability to psychopathology (Kelley et al., 2021; Yang et al., 2010; Young and Dietrich, 2015). Thus, sustained emotional information processing is not inherently beneficial or detrimental but may depend on the nature of the information being processed (positive or negative) (Harding and Mezulis, 2017). For example, rumination on positive things is potentially beneficial to mental health; positive rumination is sometimes, in the literature, negatively correlated with negative rumination (Feldman et al., 2008; Raes et al., 2014), although this observation has not been evaluated robustly across a wide range of rumination.

Whereas these differential outcomes could suggest that different people are prone to savor positive versus ruminate on negative information, a third literature suggests that repetitive thinking is, itself, trait-like and that it is the same people who both savor and ruminate (Segerstrom et al., 2003). Indeed, positive correlations exist between all types of repetitive thinking (e.g., depressive rumination and reminiscing) regardless of their content or outcome on well-being (Evans and Segerstrom, 2011; Watkins, 2008), although much of the work positively correlating periods of positive and negative affect has relied on retrospective, self-reported measures which may be subject to recall bias or other related biases (Watkins, 2008). A different, consistent literature suggests that hedonic capacity (Saxena et al., 2017) involves the tendency or ability to engage with emotion states, regardless of affective intensity. Conditions, such as dissociation and feeling numb, are posited to involve increased regulatory control yielding decreased affective intensity writ large (Frewen and Lanius, 2006). The conclusion, from both of these literatures is that, potentially, the same people will evidence increased reactivity to positive and negative prompts, associated with similarly disinhibited and sustained brain reactivity.

This observation poses questions about the mechanistic and phenomenological independence of sustained positive and negative thinking and whether they can be independently manipulated. In many disorders in which negative rumination is a detrimental symptom (e.g., attachment disorders, binge eating disorders, depression, etc.), treatments include steps to decrease negative ruminatory tendencies (Dingemans et al., 2017; Li et al., 2018a; Quickert and MacDonald, 2020). If positive and negative repetitive thinking are linked, the effectiveness of and compliance with such strategies may be partially dependent upon an individual’s trait repetitive thinking patterns. Evidence on this issue is lacking, so it is prudent that more work be done to uncover possible interconnectedness between these two types of repetitive thinking.

Phenomenological models of emotion could inform how savoring and rumination are likely to be related. For example, the popular “circumplex model” posits that negative and positive affect, at least as they occur instantaneously, are oppositionally related via a valence axis with positive and negative affect as the two poles (Barrett and Russell, 1999), and an orthogonal arousal axis, with independent brain systems for each axis, which combine to produce emotion (Posner et al., 2005; Russell, 1980). Other models, such as the Evaluative Space Model (Norris et al., 2010), consider positive and negative affect to be orthogonal, yielding a space in which both positive and negative affect can coexist and be related toward affective behaviors, such as approach. Both models could accommodate trait-like phenomena, such as predisposition toward repetitive thought, reflecting activity in common brain regions producing positive and/or negative affect. For example, differential processing of reward versus loss cues appears to be a factor responsible for the different outcomes in those with trait rumination while activity of shared brain regions is positively associated with general rumination (Kocsel et al., 2017).

Formulations suggesting that aspects of positive and negative affect are related are echoed in neuroscience, with common brain systems supporting both positive and negative affect. For example, the brain’s “salience network” is associated with detection of emotional information and generation of feeling, regardless of the information’s valence (Anikin, 2020). It contains regions, such as the insula and amygdala, which respond to any emotional information (Menon and Uddin, 2010; Sergerie et al., 2008). Similarly, the brain’s reward network is associated with responses to both reward and punishment. Regulatory brain networks, such as the “task” and “executive control” networks, particularly involving prefrontal cortical regions, are identified with regulation of both positive and negative affect (Etkin et al., 2011). Few brain networks appear to respond differentially to positive and negative affect; rather, classifying affective valence based on brain activity is known to be complicated (Berridge, 2018). This is potentially because common modules evaluate the full range of affective characteristics of a stimulus. Finally, prefrontal regulatory mechanisms appear to be involved in the down-regulation or inhibition of both positive and negative emotion (Li et al., 2018b; Ochsner et al., 2004). Whether such systems would support trait-like predispositions to settle on one valence (e.g., suggesting those who ruminate on negative information are less likely to savor positive information) or whether more active affective-evaluation modules would suggest higher trait-like predispositions to both savoring and rumination is unclear.

These observations, which indicate a potential interconnectedness between the experiencing and processing of positive and negative affect, lead to the theory that the same brain mechanisms are in place for processing positive and negative information. Thus, trait variation in the experience of one is likely to be reflected in the experience of the other, rather than simply biased processing. With emotional salience being an evolutionarily significant quality to compute for survival (Nyklíček et al., 2007), common brain mechanisms would be efficient for computation of emotional salience regardless of valence. In such a system, although the same individuals might tend, in their everyday lives, toward biased positive or negative processing, given that the systems have time to settle in one state or another, in a lab-based provocation design, we hypothesized that those individuals who would ruminate more intensely also would tend to savor more intensely.

To explore this theory, the current study used fMRI to observe participant neural activity and a real-time, bipolar, continuous, self-report scale, which measured participant mood while repetitively engaging with either positive (savoring) or negative (ruminating) thoughts. This design sought to enable the pairing of subjective mood data with fMRI data to examine associations between the valence and intensity of participant mood and neuronal activity in the brain.

To supplement this investigation, it was important to consider mechanisms that could jointly affect sustained positive and negative affect. Two literatures seemed particularly relevant for this purpose: sleep and memory specificity. Sleep disturbances and sleep loss are broadly associated with complex alterations across multiple facets of emotion regulation (Palmer and Alfano, 2017). Some observed consequences include decreased positive affect (Bower et al., 2010) but increased neural reactivity to positive experiences (Gujar et al., 2011) as well as increased negative mood and rumination (Lo et al., 2016; Thomsen et al., 2003). The relationship between sleep and savoring has been relatively less examined, and limited findings are equivocal (Tighe et al., 2022).

Memory specificity refers to the ability of individuals to recall particular events from their lives. Patients with depression have difficulty recalling specific memories and instead recall overgeneral memories, which involve more abstract or conceptual knowledge without reference to one particular event (Conway and Bekerian, 1987; Williams et al., 1986). Tendency toward memory specificity functions in a related manner to sleep; increased overgeneral memory is associated with a bias against positive memories and positive affect as seen in people with major depressive disorder (Young et al., 2013), consistent with observations of increased memory specificity being associated with higher positive affect and less negative affect (Rubin and Berntsen, 2003). That said, other data suggest that low memory specificity is associated with less subjective distress upon remembering negative autobiographical memories (Raes et al., 2003, 2006), consistent with memory specificity being associated with activity in brain systems, such as the salience and self-relevance networks, which are involved in generation of both positive and negative emotions (Barry et al., 2018). Trait-level associations of memory specificity with rumination are not observed (Chiu et al., 2018), suggesting that repetitive emotional thinking and memory specificity may act orthogonally. Thus, measures of sleep quality and memory specificity were correlated with both dynamic mood ratings and associated brain data for this study.

Consistent with calls to do more dynamic assessment of sustained processes, such as savoring (Bryant, 2021), we had four primary questions stemming from this framework, which were addressed by using subjective dynamic affect ratings, and fMRI from 60 participants, stratified on levels of self-reported sleep disturbance, who performed a task that alternated between instructions to savor or ruminate while rating their emotional intensity. *Q1) Do the same individuals who have increased emotional intensity during savoring also have increased emotional intensity during rumination?* We hypothesized that the same participants who experienced increased emotional intensity during savoring also would experience increased emotional intensity during rumination. *Q2) Are the brain processes associated with savoring and rumination similar or different?* We hypothesized that there would be mechanistic similarities between the processes of savoring and rumination. We also hypothesized that the same people who have high activity in commonly activated regions during savoring would have high activity in these regions during rumination. *Q3) Were the effects of memory/thought specificity on emotional intensity similar or different for savoring and rumination, and did memory/thought specificity for savoring and rumination correlate across individuals?* We hypothesized that participants with specific memories/thoughts would experience a greater degree of emotional intensity during periods of both savoring and rumination compared with those with less specific memories/thoughts. Exploratory analyses examined the role of specific memories in neural reactivity to savoring and rumination. *Q4) Did sleep quality play similar or different roles in emotional intensity in response to savoring and rumination?* We hypothesized that participants who reported poor quality sleep would be inclined to experience a greater degree of negative affect during periods of rumination; literature could be seen to support levels of positive affect during savoring that were either higher (due to sleep effects on common mechanisms) or lower (due to sleep effects on valence specific mechanisms). Additional exploratory analyses examined associations between multiple sleep characteristics based on sleep diary and wrist actigraphy and the neural reactivity to savoring and rumination.

## Methods

### Participants

This study was conducted within a larger project examining the impact of sleep characteristics on cognitive and emotional information processing (R21 MH102412). As such, a clinical interview and measures of sleep and mood were utilized to select 76 subjects without a psychiatric diagnosis, aged 18-30 years (36 males and 40 females; 12 African American, 49 white, 15 other, and 6 Hispanic; right-handed only) to sign informed consent. Sixty participants (29 males and 31 females; 11 African American, 37 white, 12 other, and 6 Hispanic) completed the study; ten were not eligible after the clinical interview, and six withdrew before completion. Participants were recruited to reflect a continuum of self-reported sleep disturbance based on the PROMIS Sleep Disturbance (PSD) scale, with the goal of having ~20 participants each with scores that were low (i.e., good sleep; PSD scores ≤45; *n* = 21), middle (i.e., average sleep; PSD scores 46–55; *n* = 19), and high (i.e., poor sleep; PSD scores >55; *n* = 20). Participants were required not to have significant or unstable acute or chronic medical conditions, not have current major syndromal psychiatric disorders based on a SCID-IV interview, not have current sleep disorders other than insomnia, not use medications known to affect sleep or wake function, or have any contraindications for functional magnetic resonance imaging (fMRI). The study activities included actigraphy and sleep diaries, questionnaires, and an fMRI scan. The research study was reviewed and approved by The Review Board of The University of Pittsburgh, and all subjects provided written, informed consent to participate. Recruitment of participants used advertisements on participant registries (Pitt+Me) and on social media as well as through word-of-mouth marketing. Our participant flow is illustrated in Supplement 1, Figure S1. Participants received up to $250 for their participation.

### Clinical and self-report measures

#### Structured Clinical Interview for the Diagnostic and Statistical Manual, Fourth Edition (SCID-IV) (First & Spitzer, 1996)

Exclusion diagnoses were established via the SCID-IV, a semistructured interview that follows DSM criteria, administered by Ph.D.-level clinicians.

#### PROMIS Sleep Disturbance (PROMIS-SD)

(Buysse et al., 2010; Yu et al., 2012) is a self-report measure that assesses sleep disturbance over the past 7 days. Scores were obtained from the Assessment Center (https://www.assessmentcenter.net/), which uses Computerized Adaptive Testing to obtain a standardized T-scores (mean of 50 and standard deviation of 10). Items include assessment of difficulty falling and/or staying asleep, restlessness, satisfaction with sleep, sleep amount, and sleep quality.

### Procedure

#### Study context

fMRI data in this protocol were collected as part of a multiweek study in which sleep diaries and actigraphy were assessed over ~1 week (not analyzed here), followed by an approximately 2-hour fMRI assessment in which the savoring/rumination task described below and an emotion regulation task were counterbalanced, followed by three other functional tasks. Only the savoring/rumination task is analyzed in this manuscript.

#### Savoring/rumination task

**Fig. 1:**
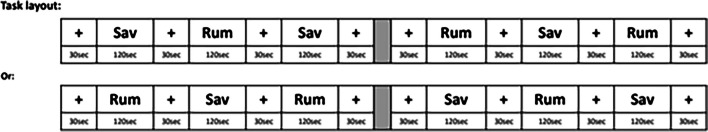
Temporal flows for the administered Savoring (Sav)/Rumination (Rum) task

During fMRI assessment, participants received instructions to alternately savor (120 seconds) or ruminate (120 seconds) for three savoring and three rumination trials; which task came first was randomized across participants. During blocks, short vignettes generated by each participant (~10 to 20 words; instructions in Supplement 5) were presented on screen inside the following script: “ruminate on something negative. Your negative script was…” or “Savor something positive. Your positive script was…”. These blocks were broken up with no-instruction fixation blocks (30 seconds) (Fig. 1).

During the savoring and rumination trials, participants were asked to rate their moods continuously on a bipolar rating scale with anchors for very sad, somewhat sad, neutral, somewhat happy, and very happy by using an MRI-compatible mouse. Affect ratings were stored at 1/second throughout the 16-minute (960 second) task.

#### fMRI acquisition

For functional imaging, 51 image slices were collected with an AC-PC alignment to acquire the regions and networks of interest. The sequence (HCP multiband, Grappa 3, TR = 1,500 ms, TE = 35 ms, FOV = 22 cm, flip = 58, 96 x 96 x 51 2.29- x 2.29- x 2.3-mm voxels, on a 3T Siemens Prisma scanner) permitted acquisition of an entire image, including the frontal, temporal, and parietal regions, every 1.5 seconds. Participants lay supine in the scanner, where a mirror was used to see a projection screen for the tasks outside the scanner. Continuous ratings were recorded with FOM-2B-10B fMRI Mouse System from NATA technologies (*Website*, n.d.). In addition, high density structural images (1 mm^3^ axial MPRAGE) were collected for cross registration.

#### fMRI preprocessing

Functional images were preprocessed using AFNI’s proc.py (Cox, 1996), including slice time correction (to slice 0, bottom of the brain), motion correction, quadratic detrending, voxelwise despiking, conversion to percent change, temporal smoothing (bandpass 0.01 to 0.1846 hz as recommended, for this TR, by Worsley and Friston (1995), to reduce high frequency artifacts), nonlinear cross-registration to the Montreal Neurological Institute Colin-27 reference brain, and spatial smoothing (4-mm FWHM) within that mask. Default options for AFNI’s proc.py were used for all options not explicitly described. For each participant, voxelwise regressions were conducted for periods of fixation, savoring, and rumination, convolved with a canonical double-gamma hemodynamic response to yield beta weights for neural reactivity in these periods. Voxelwise beta-weight outliers were Winsorized (rescaled to the Tukey hinges (last valid value within 1.5*IQR from the 25th and 75th percentiles) to yield robust estimates of neural reactivity per participant.

### Analysis methods

All tests used all 60 participants; complete data on all participants were available for all tests.

#### Q1 Subjective affect ratings

To examine whether the same participants experienced increased emotional intensity during periods of both positive and negative affect, continuous affect ratings were averaged within the savoring, rumination, and fixation conditions. Following samplewise analyses to make sure there were not critical temporal regions of significant differences, mean condition-related affect ratings were subjected to an ANOVA followed by a *post-hoc* Dunn’s multiple comparisons test to examine differences in affect during the three trial types. Convergence of affect ratings in the savoring and rumination conditions was examined by using a Pearson correlation test.

#### Q2 Neural reactivity

To observe the brain mechanisms associated with savoring and rumination, beta-weight masks from single-subject voxelwise regressions were compared across participants by using paired *t*-tests for condition related differences, in an effective multilevel hierarchical linear model (preserving means, but not variances from the single-subject models), evaluated at voxelwise *p* < 0.005, subjected to empirical clustering thresholding to control type I error at *p* < 0.05 via permutation testing within AFNI’s 3dTtest++ routine. Voxelwise correlations were used to establish associations in reactivity between the tasks.

#### Q3. Memory specificity

The effect of memory specificity was observed using participants’ three positive and three negative vignettes used as prompts for the savor and rumination trials. Each vignette was coded for specificity according to standard definitions used in the AM literature (Williams et al., 2007; Williams & Scott, 1988; Anderson & Levy, 2009) (details in Supplement 6). All responses were rated by one rater (KY), and an independent rater scored 39% of responses to establish interrater reliability (agreement = 89%, Cohen’s k = 0.83). Mixed effects models were examined with each memory coded as a repeated measure (3 per subject per valence) to determine whether specific memories during periods of savoring were associated with more positive affect, whether specific memories during rumination were associated with more negative affect, and whether the same participants displayed increased specificity to savoring as rumination. Finally, a chi-squared test was performed to determine whether more specific memories were present during periods of savoring compared with rumination. Voxelwise simultaneous regression was used to examine relationships of rumination and savoring memory specificity to fMRI-derived beta-weight maps for rumination and savoring. Because of a wide literature associating memory specificity with activity in small structures, such as the amygdala and hippocampus (Chavez et al., 2013; Tanaka et al., 2014), exploratory analyses employing a priori contiguity threshold of 100 voxels was used to allow detection of such regions, with intent to report specifically if activations were found in the neighborhood of these medial-temporal structures.

#### Q4. Sleep quality

Sleep quality, assessed by using the PROMIS sleep disturbance scale as our primary measure, was correlated with mean affect ratings during the savoring and rumination conditions. Voxelwise regression was used to associate sleep quality with fMRI-derived beta-weight maps for rumination and savoring.

#### Sample size justification

Consistent with our field’s general guidelines regarding the utility of benchmark targets, rather than empirically based effect sizes (Kraemer & Kupfer, 2006), and modality-specific findings, primary analyses, were powered for small effects in subjective affect and large effects in fMRI, such that with *N* = 60 and power >0.8, for zero-order correlations between indices of rumination and savoring we could detect correlations as small as r = 0.345 at *p* < 0.05, and voxelwise condition-related differences in association with reactivity to rumination and savoring as small as Cohen’s d = 1 at *p* < 0.005.

#### Data availability

Examined single-subject indices and raw data are available from the contact author upon request along with our code for preprocessing and analyzing that data as described in this manuscript.

#### Preregistration

Questions addressed in this manuscript were not preregistered.

## Results

### Behavioral manipulation check

**Fig. 2:**
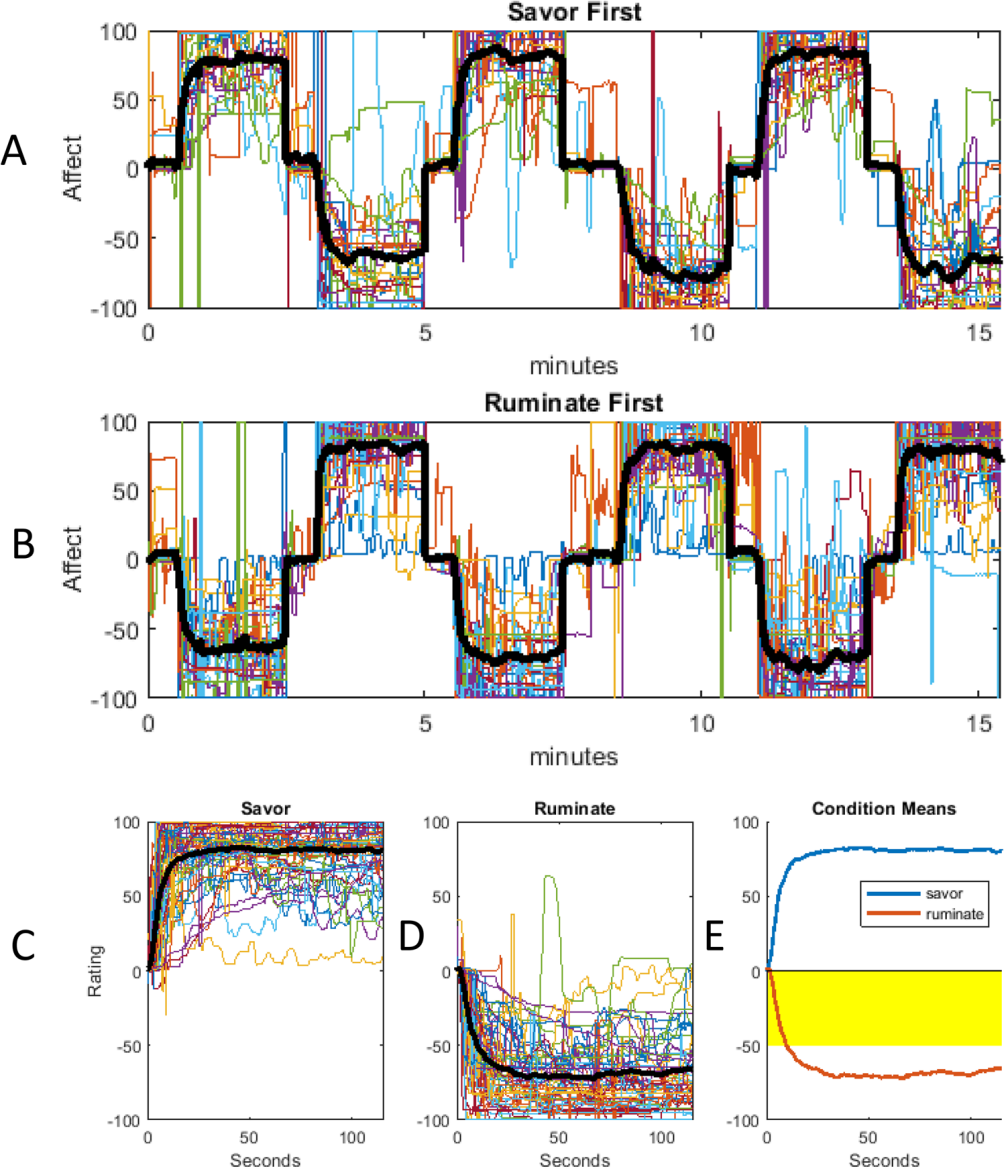
Continuous participant affect during administration of savoring/rumination task. **A**, **B** Each participant’s continuous affect ratings throughout the task (1 value per second) are shown with the mean of these ratings highlighted in black. **A** Data from participants who savored first. **B** Data from participants who ruminated first. Data are presented in mouse ratings, where 0 represents neutral affect, 100 represents the highest possible positive affect, and −100 represents the greatest possible negative affect. **C**, **D** Condition-related averages for savoring and rumination for each participant, showing that generally, participants’ affect increased and stayed high during savoring, and decreased and stayed low during rumination. **E** Contrast of condition-related reactivity for savoring vs. rumination. The shaded area below the axis represents regions of statistically significant differences, *p* < 0.05

**Fig. 3:**
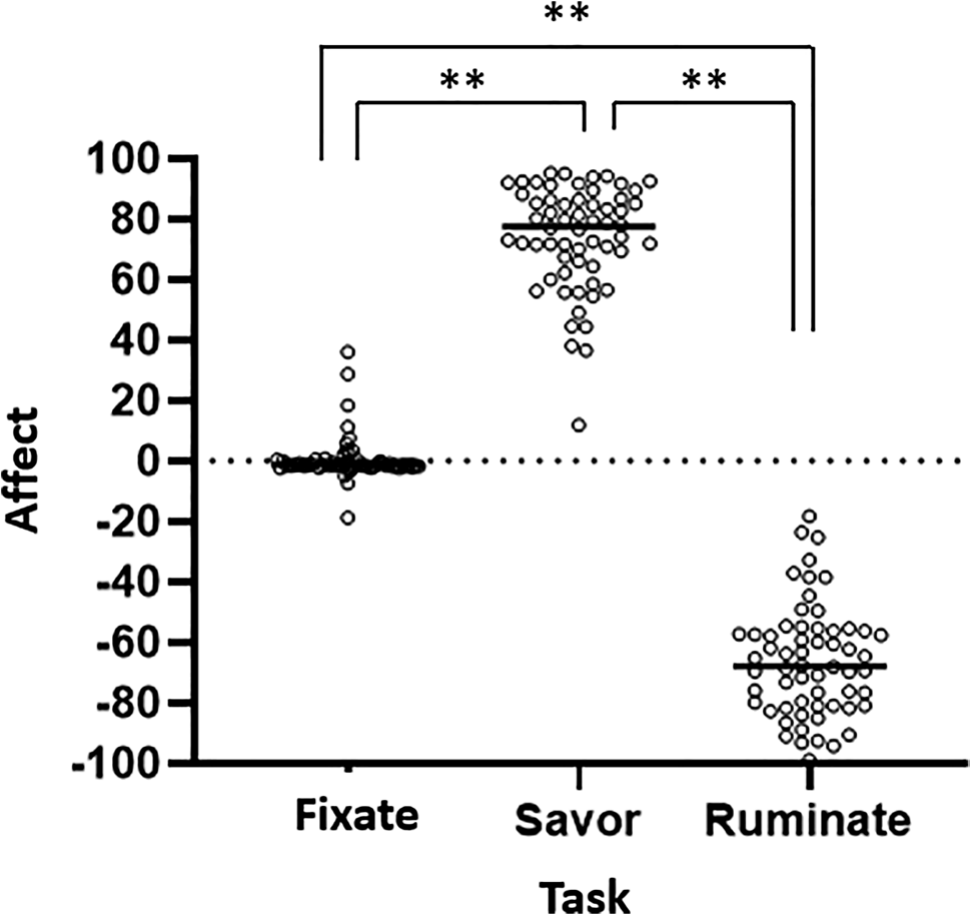
Mean participant affect during components of Savoring/Rumination task with black bars for group condition means. 0 = neutral affect; 100 = greatest positive affect; −100 = greatest negative affect. **p < 0.005

Participants’ affect generally increased (toward positive valence) during savoring and decreased (toward negative valence) during rumination (Fig. 2) with differences in mean condition-related affective ratings being significant throughout the entire time-course, from 0.8 to 120 seconds, F(1,59) = 1145.65, *p* < 0.005, R^2^ = 0.91. A Mann-Whittney test, α = 0.05, followed by a *post-hoc* Dunn’s multiple comparison’s test, revealed that mean affect ratings were significantly different between each of the fixation, savoring, and rumination tasks, all *p* < 0.005 (Fig. 3).

### Q1. Relationships between savoring and rumination reactivity

**Fig. 4:**
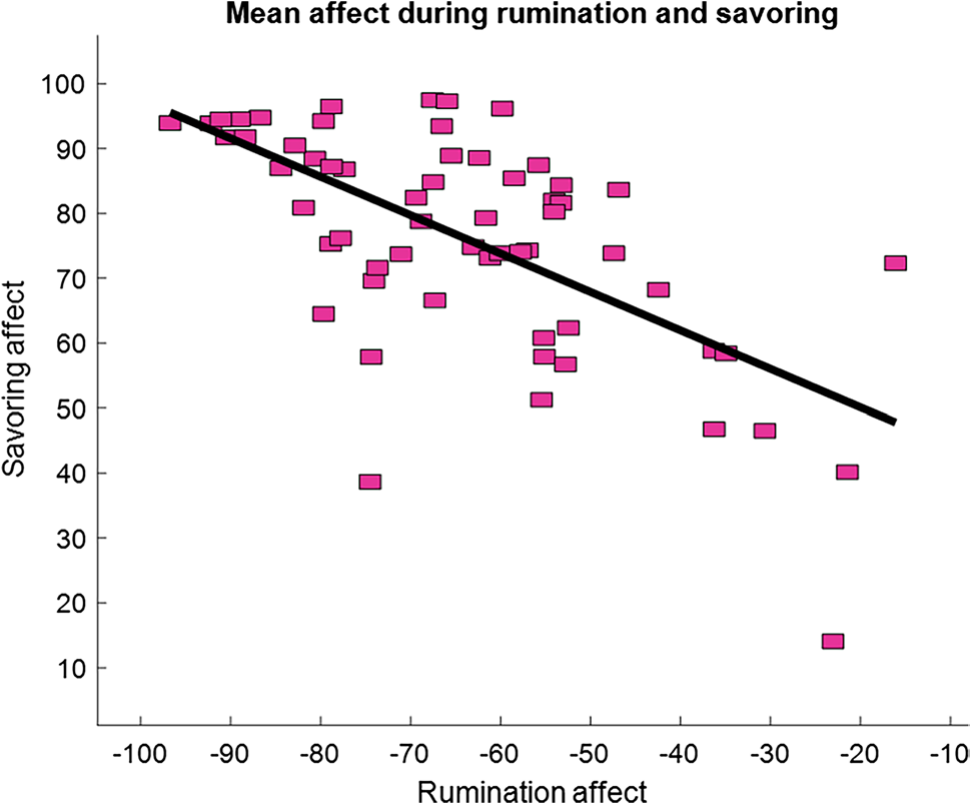
Mean participant affect for Savoring and Rumination components of Savoring/Rumination task. 0 = neutral affect; 100 = greatest positive affect; −100 = greatest negative affect

Individuals whose affect was more positive during savoring also experienced more negative affect during rumination, r = −0.63, F(1,59) = 38.7, *p* < 0.0001 (Fig. 4).

### Q2. Neural reactivity during savoring and rumination

**Fig. 5:**
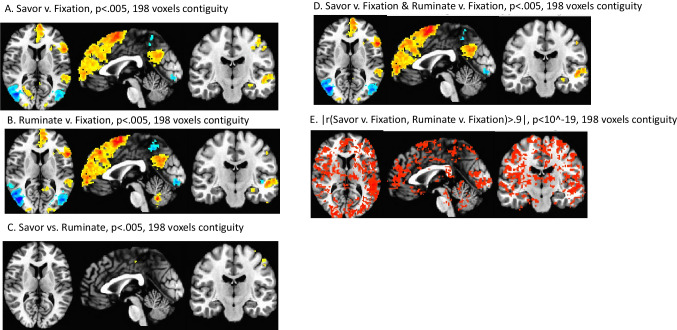
fMRI results: Voxelwise BOLD responses to condition-related contrasts on beta weights from single-subject regressions, p < 0.005, 198 voxels contiguity threshold, empirically determined.** A**, **B** Contrasts for rumination and savoring vs. fixation. **C** Differences in activity between the savor and rumination tasks. **D** Conjunction of A, B. **E** Correlation of savoring and rumination beta weights, |r| > 0.9 to emphasize the very strongest correlations; nearly the entire brain was correlated with |r| > 0.8

As shown in Figs. 5A, B, C, D, nearly the same networks were activated for savoring and rumination, primarily including the brain’s default, executive, salience, and memory networks (e.g., amygdala/hippocampus, frontal polar regions, peri-genual cingulate cortex, dorsolateral prefrontal, and bilateral parietal regions). Differences between the conditions were minimal compared to overlapping areas and were confined to motor regions (see Supplement 2 for subthreshold differences). Almost all gray matter showed strong positive correlations (r > 0.8) between mean activation during savoring and rumination, across participants. Figure 5E shows that much of the brain displayed correlations r > 0.9. Region-wise centroids, Talairach coordinates, and which atlas regions are covered by each are listed in Supplement 2. Supplement 2 also notes that these maps were not strongly associated with behavioral rating magnitudes for savoring and rumination.

### Q3. Specific memories

Participants who had more specific memories during savoring trials (mean across blocks) were also more likely to have more specific memories during rumination trials, r = 0.29, *p* = 0.027. Mixed-effects analysis across sessions, using session as a repeated measure with an AR(1) covariance structure, memory specificity as an independent variable, and mean affect as the dependent variable suggested that the presence of specific memories were not significantly related to mean affect during savoring, F(1,138.21) = 0.049, *p* = 0.83, with a low correlation of mean savoring and specificity across blocks r_across_blocks_ = 0.15, *p* = 0.25. A similar lack of a relationship was present for rumination, F(1,157.41)=1.51, p=0.22, r_across_blocks_=-0.11, p=0.42. Participants reported more specific memories during savoring than rumination trials, M(SD) difference = 0.28(1.01) memories, t(59) = 2.17, *p* = 0.034, Cohen’s d = 0.34. There were no significant associations of specific memory frequencies and brain reactivity during savoring or rumination, which survived cluster thresholding at *p* < 0.005. Given previous literature associating amygdala reactivity with specific memories, a less stringent threshold and smaller cluster value were examined to allow inferences about such smaller anatomically constrained regions; indeed similar nonsignificantly large hippocampal/amygdala regions were associated with both savoring and rumination (Supplement 3).

### Q4. Sleep quality

**Fig. 6:**
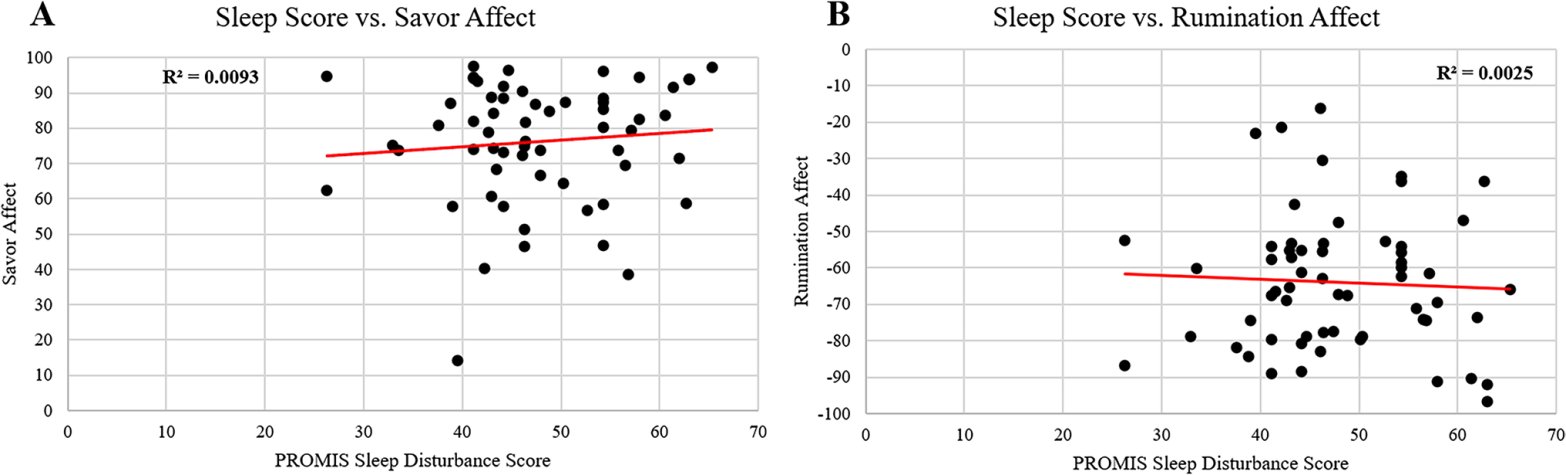
Relationship between participant sleep quality (PROMIS sleep disturbance) and mean subjective affect during **A** Savoring and **B** Rumination (*p* > 0.4).

PROMIS Sleep Disturbance scale scores were not significantly correlated with mean affect across blocks of savoring, r = 0.096, F(1,59) = 0.54, *p* = 0.46, or rumination, r = 0.05, F(1,59) = 0.14, *p* = 0.70 (Fig. 6). There were no significant associations of sleep quality and brain reactivity during savoring or rumination which survived cluster thresholding at *p* < 0.005. As part of an exploratory, supplemental analysis undertaken to prevent Type II error, a nonsignificantly large region of the right inferior parietal lobule (44 voxels) was associated with both savoring and rumination (shown in Supplement 4).

#### Other individual differences

Supplement 7 lists correlations of mean affect during savoring and rumination with a host of likely individual difference measures associated with trait rumination, worry, and savoring, as well as depression and anxiety. As noted in the supplement, none of these associations were statistically significant. Supplement 8 specifically tests the potential that correlations between ratings during the rumination and savoring periods were artificially inflated by individual differences in the tendency to use, or not use, the full range of rating responses. We show that this is unlikely given low correlations of mean scores with variances within conditions (Table S.8.1), but given that associations of mean scores with variances across conditions would emerge even in random data (Table S.8.2), the design is not optimal for evaluating this possibility.

## Discussion

This study examined relationships between subjective affect and neural reactivity during savoring and rumination. Results suggested that the same individuals who experienced more intense positive affect during savoring also experienced more intense negative affect during rumination. The underlying neural mechanisms of these processes appeared similar, with the same individuals who had higher reactivity during savoring also having higher reactivity during rumination. The same individuals who had more specific memories during savoring also had more specific memories during rumination, but neither specific memories nor sleep moderated the behavioral association above. These results suggest that engaging with both positive and negative affect appears to be similar in individuals who do not have a psychiatric diagnosis, subjectively and mechanistically, such that the same people who are able to do one are often likely able to do the other.

Neural reactivity to both savoring and rumination spanned multiple commonly implicated brain networks including the default mode network (multiple mid-line structures including the frontal pole, rostral cingulate, and posterior cingulate), salience network (e.g., amygdala), and memory network (e.g., hippocampus), and both positively and negatively with different portions of the executive network. These networks are consistent with those observed in other neuroimaging studies of rumination (Kühn et al., 2012; Makovac et al., 2020; Mandell et al., 2014; Zhou et al., 2020). That they also apply to savoring suggests that potentially, much of the current mechanistic understanding of rumination also may be applied to savoring of positive information. That is, regions and networks underlying rumination and savoring may be shared; although the computations and types of processing these networks do to arrive at valence-specific outcomes may differ, correlated affect ratings across savoring and rumination also argue for shared processes at this level. Indeed, while savoring and ruminating differ in their valence and consequent theoretical behavioral functions, the literature shows that the same brain networks, particularly those involving the striatum, are associated with both reward and punishment (Delgado et al., 2000; Metereau & Dreher, 2013). With this in mind, it is not surprising that individual differences have been demonstrated to govern whether individuals are highly or minimally responsive to both rewards and punishments (Boksem et al., 2006; Kim et al., 2015).

The ecological validity of the current experimental procedure is undetermined. It is possible that real-life savoring and ruminating manifest in ways which are not simulated by the current procedure. Additionally, the subjective intensities of affect were not strongly associated with neural map intensities during periods of either savor and rumination. That said, there is substantial work showing that cross-modality associations of this nature are often weak, in part due to the measurement error associated with each respective modality (Mauss et al., 2005). Thus, associational studies involving neural and behavioral components likely need substantially larger sample sizes to produce consistent, detectable results (Marek et al., 2022).

Increased specific memories appeared to contribute to neural reactivity associated with both savoring and rumination in expected amygdala and hippocampal regions, but this did not translate to behavioral associations. It is, however, interesting to note that despite there being no instructions to develop specific memories, specificity during periods of savor and rumination were correlated, which indicates a stable response style.

Surprisingly, sleep quality did not appear associated with mechanisms of savoring or rumination, potentially due to the nonclinical nature of the population and the lack of task demands (e.g., stressor or dual task), which could have provoked sleep-related disturbances in affective processing. We acknowledge that we did not preregister our question and that the parent study from which our data came had hundreds of variables, which were irrelevant to our questions. As such, we took care to consider a broad subset of potentially relevant variables, none of which were significant (Supplement 7) to ensure that we had observed as much potentially relevant data as possible.

Ultimately, this study was designed to explore associations at the level of form, not function. The theory that common brain mechanisms developed to compute emotional salience regardless of valence, which we proposed in the introduction, is at its core evolutionary and unable to be tested causally, although our data are consistent with that theory.

As such, we have limited our conclusions to features that we could test. Our results have basic implications and, to the extent that they generalize to clinical populations, potential implications for clinical practice. On a basic level, our findings have implications for how we understand affect and whether we think of positive and negative affect as mechanistic opposites. Indeed, our results are consistent with other work that suggests that the same brain structures are responsible for reward and punishment (Carter et al., 2009; Shigemune et al., 2014) and responses to positive and negative emotional stimuli (Kensinger & Schacter, 2006). Expanding that understanding to suggest that preserving the mechanistic capacity for intense negative emotions also may be important to preserving positive emotions is a logical next-step. Our results begin to call into question where lines between functional concepts like affective range end, and more stigmatized concepts, such as affective lability, begin (Hawke et al., 2013; Lazowski et al., 2012). They also promote consideration of movement beyond the bipolar affective circumplex to representations that allow for convergence in positive and negative affect, more like the evaluative space model (Norris et al., 2010), or a three-dimensional polar coordinates model in which positive and negative affect could appear quite different at low levels of arousal (near the “equator”) but converge at higher levels of arousal (near the “north pole” yielding convergence, e.g., of rapture and despair).

If these results generalize to clinical populations, this formulation could explain why pharmacological treatments that diminish negative affect (e.g., SSRI’s) also are reported to diminish positive affect (Goodwin et al., 2017; Opbroek et al., 2002; Sansone & Sansone, 2010), suggesting utility for strategies that help to intentionally regulate, rather than simply diminish, emotion. It also could suggest that therapies that aim to instill automatic emotion regulatory responses for affect could cut both ways if care is not taken to permit and amplify positive affect, even in the presence of novel regulation of affect. Indeed, our data are further consistent with the idea that individuals who ruminate may have preserved capacity for savoring and that the tendency for sustained processing of emotional information, rather than being dampened, could potentially be co-opted to balance affective reactivity by also savoring positive information. Such a recommendation is consistent with the recent change to the canonical Cognitive Therapy texts, which had almost exclusively concentrated on diminishing negative affect, to now emphasize preserving and amplifying positive affect (Beck, 2020). We have found that individuals who are dysphoric are not only able to savor, but when instructed to do so, experience reductions in depressive symptomatology (McMakin et al., 2011).

This study had multiple limitations. The N was relatively small and not powerful enough to detect smaller effects, such as those examined in Supplements 2 and 4, which may account for subjective differences in how savoring and rumination feel. Potential clinical implications are particularly speculative as only young (18-30 years old) adults without a psychiatric diagnosis were assessed. Replication in clinical populations could be useful, as individuals expected to ruminate most were not included in this study.

Despite our instructions, participants may have interpreted the instructions to savor and ruminate differently from each other such that there were differences in the characteristics of each individual’s thoughts during these tasks. Fortunately, the definitions for savoring and ruminating which guided the current study were aligned with those used in the Response Styles Questionnaire (measure of rumination) and the associated Responses to Positive Affect Scale (measure of savor). These measures are written from the perspective in which rumination is operationally defined as thinking about one’s negative emotions and savoring is operationally defined as thinking about one’s positive emotions which allows for some flexibility in the individual’s engagement with either savoring or ruminating.

The extent to which participant insight into the emotional component of the task, continuous affect ratings, and the switching demands of the current task were disruptive to savoring and rumination was unclear. Concerns about these potential confounds are prevalent in affective science studies which rely on self-report measures of emotion, contiguous emotional ratings, and randomized emotional conditions. Thus, the current study acknowledges the same potential limitations as those of these related works. Fortunately, previous experiments designed to detect disruptive effects in response to continuous affective stimuli have found none (Hutcherson et al., 2005). The implementation of this bipolar rating scale occurred before our theoretical framework for the current study was developed. While the use of a bipolar dynamic rating scale for affect could have obscured more independence between positive and negative affect, we opted to keep this bipolar approach due to the complexity of participants using a self-report measure with a dynamic rating scale and multiple dimensions to capture a more comprehensive view of the broad range of emotions accessible through self-report measures (Cowen & Keltner, 2017). The higher effort needed to rate such a scale could have potentially reduced the level of affect associated with the task. We thus opted for potential measurement error, with respect to our framework, over the potentially invalidating effects of effortful ratings. However, given the current results, replication with such techniques may be warranted.

The fixed order of savoring alternating with rumination yielded the potential that rumination could affect subsequent savoring or vice versa. Indeed, a behavioral study using a similar task but with variation in block orders (Cummings et al., submitted) suggested that, at least in depressed adolescents, savoring following a neutral context yielded different behavioral ratings than savoring following rumination. Furthermore, our hope was that the use of the fixation period, in which participants also rated their emotional valence, as a contrast from periods of savoring and ruminating, negated such task effects.

These limitations notwithstanding, we suggest, based on the current experiment, that savoring and rumination be considered opposite sides of the same coin and that convincing data suggest the same individuals who ruminate also may have preserved and potentially enhanced capacity for savoring, which could, pending replication in clinical samples, lead to targeted application of savoring-related intervention strategies for disorders characterized by perseverative negative thinking. We hope to conduct future studies that investigate this same question in clinical populations and explore the role of individual participant differences in the neural and subjective relationships between savoring and ruminating.

Raw and processed data as well as summary indices are available from the authors by request. The experiment was not preregistered.

### Supplementary Information

Below is the link to the electronic supplementary material.

Supplementary file1 (DOCX 2.63 MB)

## Data Availability

Data have not been uploaded to a repository as our consent forms did not allow for this possibility.
