# Correction to: Subjective and neural reactivity during savoring and rumination

**DOI:** 10.3758/s13415-023-01145-w

**Published:** 2024-02-20

**Authors:** Benjamin O. Brandeis, Greg J. Siegle, Peter Franzen, Adriane Soehner, Brant Hasler, Dana McMakin, Kym Young, Daniel J. Buysse

**Affiliations:** 1 Dickinson College, Carlisle, PA USA; 2 School of Medicine, University of Pittsburgh, WPH, 3811 O’Hara St, Pittsburgh, PA 15213 USA; 3 Florida International University, Miami, FL USA

**Correction to : Cogn Affect Behavior Neurosci. 2023;23:1568-80**

10.3758/s13415-023-01123-2

The original article has been corrected to update author's name as Adriane Soehner, which was misspelled as Adriene Soehner.

