# Supplementary material for: Subjective and neural reactivity during savoring and rumination
Source: Cogn Affect Behav Neurosci. 2023 Sep 19;23(6):1568–80. doi: 10.3758/s13415-023-01123-2 (PMC10684651; doi:10.3758/s13415-023-01123-2)
Supplement: Supplementary file 1 — Supplementary file1 (DOCX 2.63 MB) [file 13415_2023_1123_MOESM1_ESM.docx]

Supplementary Information

Subjective and neural reactivity during savoring and rumination

Benjamin O. Brandeis, Greg J. Siegle, Peter Franzen, Adriene Soehner, Brant Hasler, Dana McMakin, Kym Young, Daniel J. Buysse

# Table of Contents

[**Supplement 1. Participant Flow**](#_7ys7131yardp) **2**

[**Supplement 2: Supplementary imaging data for savoring and rumination contrasts.**](#_om8en8aably1) **3**

[S2.1 Regions that activated for both savoring and rumination](#_dpjebf28vpva) 3

[S2.2 Associations of regions with behavioral data](#_qsr1u9vpjh3y) 5

[S2.3 Subthreshold regions for Savoring vs. Rumination](#_doyfado2twou) 6

[**Supplement 3: Sub-cluster-threshold regions associated with specific memories.**](#_rg1gvcnvnwta) **8**

[**Supplement 4: Sub-threshold associations with Sleep**](#_hcpu46ob30ne) **9**

[**Supplement 5: Instructions for generating scripts for the Savoring and Rumination task**](#_qs31rlej88mh) **11**

[**Supplement 6: Details of Memory Specificity Coding**](#_e60vc1fe6atm) **13**

[**Supplement 7: Association of individual differences with affect intensity ratings**](#_x75b9hl9pmxs) **14**

[S7.1 Methods](#_i6ywyf6ykg8g) 14

[S7.2 Results](#_8x0x1vow7wul) 15

[**Supplement 8: Associations of subjective rating means and range-measures**](#_g00oy3i6jwse) **16**

[**References for Supplement**](#_n5286sa42pw9) **18**

# Supplement 1. Participant Flow

Of approximately 2005 total contacts, N=777 participants completed our full web screening form, of whom 76 were consented, and 60 participated in the fMRI assessment with usable data. The participant flow, with reasons for exclusion at each step, is shown below.

N=777 completed the Web screening

- Potentially eligible but did not contact (e.g., because cells were already filled) 185
- Contraindications to MR scanning (pregnancy, claustrophobia, cardiac pace-maker, surgical clips, implanted metal screws, cochlear implants…) 116
- Current psychiatric disorder 114
- Sleep schedule: Habitual bedtime later than 2:00 a.m. and wake times after 9:00 a.m. 93
- Current use of medications known to affect sleep or wake function 61
- No longer interested 43
- Non-right handedness, determined by < 40 on Edinburgh 28
- Neurological or severe systemic medical illness 25
- Sleep schedule: Sleep times longer than 9 hours, less than 6 hours. 12
- Age is < 18 or > 30 11
- Too busy / time commitment 5
- Not willing to comply with protocol 4
- Difficulty scheduling 3
- Family burden 1

N=76 consented

- Too busy / time commitment 3
- Current psychiatric disorder 3
- Contraindications to MR scanning (pregnancy, claustrophobia, cardiac pace maker, surgical clips, implanted metal screws, cochlear implants…) 3
- Current sleep disorders other than insomnia 2
- Other 1
- Difficulty scheduling 1
- No longer interested 1
- Sleep schedule: Habitual bedtime later than 2:00 a.m. and wake times after 9:00 a.m. 1
- Non-right handedness, determined by < 40 on Edinburgh 1

N=60 with usable fMRI data

**Table S1.** Participant flow for the current study.

# Supplement 2: Supplementary imaging data for savoring and rumination contrasts.

## S2.1 Regions that activated for both savoring and rumination

Table S2.1 shows information for regions present in the intersection of the savoring and rumination maps from the main manuscript Figure 4D. Each table entry shows the number of voxels, the Talairach coordinates of the region’s center of mass, and the percent occupation of the ROI for each atlas region covering it by at least 5 percent.

**Table S2.1.**

ROI #-voxels [Talairach-coordinates CM-X CM-Y CM-Z] %-coverage1 ROI1… %-coverageN ROI-N

1 6176 vox [-49 6 5]: 29.7 Left Inferior Frontal Gyrus, 20.0 Left Middle Temporal Gyrus, 17.7 Left Middle Frontal Gyrus, 10.8 Left Superior Temporal Gyrus, 8.5 Left Brodmann area 21, 5.0 Left Brodmann area 47, 19.5 Left Middle Temporal Gyrus, 14.1 Left Inferior Frontal Gyrus (p. Triangularis), 7.6 Left Inferior Frontal Gyrus (p. Orbitalis), 6.3 Left Middle Frontal Gyrus, 9.0 Area 45, 6.2 Area 44, 72.5 Left Brain, 15.9 Left-Cerebral-White-Matter, 14.1 ctx_lh_G_temporal_middle, 8.4 ctx_lh_G_front_middle, 7.6 ctx_lh_G_temp_sup-Lateral, 6.0 ctx_lh_G_front_inf-Triangul, 5.7 ctx_lh_G_front_inf-Opercular, 18.1 ctx-lh-middletemporal, 12.6 Left-Cerebral-White-Matter, 9.0 ctx-lh-superiortemporal, 7.8 ctx-lh-parsopercularis, 7.8 ctx-lh-caudalmiddlefrontal, 5.7 ctx-lh-parstriangularis,

2 4857 vox [-5 36 36]: 32.1 Left Superior Frontal Gyrus, 21.3 Left Medial Frontal Gyrus, 7.5 Left Brodmann area 6, 7.2 Left Brodmann area 10, 6.0 Left Brodmann area 9, 5.8 Left Brodmann area 8, 32.6 Left Superior Medial Gyrus, 15.1 Left Superior Frontal Gyrus, 12.4 Left SMA, 9.6 Left Anterior Cingulate Cortex, 6.3 Left Mid Orbital Gyrus, 6.2 Area 6, 72.7 Left Brain, 9.7 Right Brain, 47.0 ctx_lh_G_front_sup, 12.5 Left-Cerebral-White-Matter, 7.9 ctx_rh_G_front_sup, 5.5 ctx_lh_G_and_S_cingul-Ant, 50.4 ctx-lh-superiorfrontal, 10.6 Left-Cerebral-White-Matter, 7.8 ctx-rh-superiorfrontal,

3 3201 vox [50 -60 14]: 25.4 Right Middle Temporal Gyrus, 22.2 Right Middle Occipital Gyrus, 17.7 Right Inferior Parietal Lobule, 10.0 Right Supramarginal Gyrus, 5.3 Right Inferior Temporal Gyrus, 13.8 Right Brodmann area 40, 11.9 Right Brodmann area 19, 7.5 Right Brodmann area 37, 21.8 Right Middle Temporal Gyrus, 15.3 Right SupraMarginal Gyrus, 14.8 Right Middle Occipital Gyrus, 8.1 Right Inferior Temporal Gyrus, 7.1 Right Inferior Parietal Lobule, 10.3 IPC (PGp), 8.7 IPC (PFm), 8.6 IPC (PF), 5.4 IPC (PGa), 70.9 Right Brain, 24.7 Right-Cerebral-White-Matter, 18.9 ctx_rh_G_occipital_middle, 15.0 ctx_rh_G_pariet_inf-Supramar, 11.6 ctx_rh_G_pariet_inf-Angular, 5.0 ctx_rh_G_temporal_middle, 29.7 ctx-rh-inferiorparietal, 18.1 ctx-rh-supramarginal, 15.9 Right-Cerebral-White-Matter, 14.4 ctx-rh-lateraloccipital, 5.0 ctx-rh-middletemporal,

4 1699 vox [-38 -66 2]: 29.1 Left Middle Occipital Gyrus, 17.6 Left Middle Temporal Gyrus, 11.8 Left Fusiform Gyrus, 8.5 Left Culmen, 6.6 Left Inferior Temporal Gyrus, 14.0 Left Brodmann area 19, 11.2 Left Brodmann area 37, 27.9 Left Middle Occipital Gyrus, 20.0 Left Fusiform Gyrus, 9.1 Left Middle Temporal Gyrus, 7.2 IPC (PGp), 83.4 Left Brain, 43.1 Left-Cerebral-White-Matter, 24.0 ctx_lh_G_occipital_middle, 5.9 ctx_lh_G_oc-temp_lat-fusifor, 5.6 Left-Cerebellum-Cortex, 33.2 Left-Cerebral-White-Matter, 18.5 ctx-lh-lateraloccipital, 14.0 ctx-lh-inferiorparietal, 9.3 ctx-lh-fusiform, 5.0 Left-Cerebellum-Cortex, 5.0 ctx-lh-inferiortemporal,

5 1265 vox [-8 -50 20]: 36.7 Left Precuneus, 17.3 Left Posterior Cingulate, 16.2 Left Parahippocampal Gyrus, 9.0 Left Cingulate Gyrus, 17.4 Left Brodmann area 31, 10.3 Left Brodmann area 7, 50.4 Left Precuneus, 8.1 Left ParaHippocampal Gyrus, 7.1 Left Hippocampus, 7.0 Left Posterior Cingulate Cortex, 6.2 Left Calcarine Gyrus, 5.7 Left Cuneus, 7.9 Hipp (CA), 5.1 SPL (7M), 91.9 Left Brain, 7.6 Right Brain, 33.4 ctx_lh_G_precuneus, 29.1 Left-Cerebral-White-Matter, 11.2 Left-Hippocampus, 7.4 ctx_rh_G_precuneus, 5.5 ctx_lh_S_subparietal, 46.5 ctx-lh-precuneus, 22.8 Left-Cerebral-White-Matter, 11.3 Left-Hippocampus, 9.5 ctx-rh-precuneus,

6 897 vox [-22 -92 -5]: 31.1 Left Lingual Gyrus, 27.0 Left Inferior Occipital Gyrus, 16.6 Left Cuneus, 9.3 Left Fusiform Gyrus, 8.9 Left Middle Occipital Gyrus, 28.1 Left Brodmann area 18, 15.3 Left Brodmann area 17, 34.4 Left Middle Occipital Gyrus, 15.2 Left Inferior Occipital Gyrus, 10.4 Left Calcarine Gyrus, 6.5 Left Lingual Gyrus, 29.3 Area 18, 21.8 Area 17, 21.3 hOC3v (V3v), 5.8 hOC4v (V4), 86.5 Left Brain, 38.3 Left-Cerebral-White-Matter, 25.1 ctx_lh_Pole_occipital, 9.3 ctx_lh_G_occipital_middle, 58.3 ctx-lh-lateraloccipital, 21.5 Left-Cerebral-White-Matter,

7 695 vox [22 -92 -2]: 30.3 Right Lingual Gyrus, 23.1 Right Cuneus, 22.0 Right Inferior Occipital Gyrus, 13.7 Right Middle Occipital Gyrus, 6.5 Right Fusiform Gyrus, 29.7 Right Brodmann area 18, 11.9 Right Brodmann area 17, 24.0 Right Calcarine Gyrus, 19.9 Right Inferior Occipital Gyrus, 10.5 Right Lingual Gyrus, 8.4 Right Superior Occipital Gyrus, 6.2 Right Cuneus, 5.9 Right Middle Occipital Gyrus, 43.5 Area 17, 32.5 Area 18, 8.2 hOC3v (V3v), 82.2 Right Brain, 42.8 Right-Cerebral-White-Matter, 36.4 ctx_rh_Pole_occipital, 48.3 ctx-rh-lateraloccipital, 32.2 Right-Cerebral-White-Matter,

8 678 vox [-48 -61 28]: 27.3 Left Superior Temporal Gyrus, 27.0 Left Middle Temporal Gyrus, 23.9 Left Angular Gyrus, 6.9 Left Inferior Parietal Lobule, 6.2 Left Supramarginal Gyrus, 40.7 Left Brodmann area 39, 52.9 Left Angular Gyrus, 5.9 Left Middle Temporal Gyrus, 28.3 IPC (PGa), 22.6 IPC (PGp), 65.5 Left Brain, 52.8 ctx_lh_G_pariet_inf-Angular, 25.8 Left-Cerebral-White-Matter, 59.8 ctx-lh-inferiorparietal, 14.4 Left-Cerebral-White-Matter, 8.7 ctx-lh-supramarginal,

9 484 vox [42 19 -22]: 25.5 Right Inferior Frontal Gyrus, 20.9 Right Superior Temporal Gyrus, 15.3 Right Middle Temporal Gyrus, 5.9 Right Middle Frontal Gyrus, 13.6 Right Brodmann area 38, 13.2 Right Brodmann area 47, 9.1 Right Brodmann area 21, 32.6 Right Medial Temporal Pole, 15.8 Right Temporal Pole, 10.1 Right Inferior Frontal Gyrus (p. Orbitalis), 72.1 Right Brain, 24.2 ctx_rh_G_temporal_middle, 22.4 ctx_rh_Pole_temporal, 19.1 ctx_rh_G_orbital, 8.5 ctx_rh_G_temp_sup-Lateral, 34.8 ctx-rh-middletemporal, 16.5 ctx-rh-lateralorbitofrontal, 16.3 ctx-rh-superiortemporal, 5.0 ctx-rh-temporalpole,

10 474 vox [3 -54 55]: 55.3 Right Precuneus, 9.4 Left Precuneus, 7.7 Left Superior Parietal Lobule, 29.8 Right Brodmann area 7, 7.7 Left Brodmann area 7, 63.1 Right Precuneus, 16.5 Left Precuneus, 28.4 SPL (5M), 20.9 SPL (5L), 15.6 SPL (7A), 69.4 Right Brain, 17.7 Left Brain, 33.6 ctx_rh_G_precuneus, 17.8 Right-Cerebral-White-Matter, 11.3 ctx_lh_G_parietal_sup, 7.8 ctx_rh_G_parietal_sup, 36.2 ctx-rh-precuneus, 16.4 ctx-rh-superiorparietal, 12.1 Right-Cerebral-White-Matter, 12.0 ctx-lh-superiorparietal,

11 396 vox [29 -50 -13]: 31.4 Right Fusiform Gyrus, 23.0 Right Culmen, 23.0 Right Declive, 11.3 Right Parahippocampal Gyrus, 5.9 Right Lingual Gyrus, 15.6 Right Brodmann area 37, 65.0 Right Fusiform Gyrus, 19.0 Right Cerebellum (VI), 7.9 Right Lingual Gyrus, 5.7 Right Inferior Temporal Gyrus, 13.1 Lobule VI (Hem), 11.4 hOC4v (V4), 100.0 Brain, 43.6 Right-Cerebral-White-Matter, 21.7 ctx_rh_G_oc-temp_lat-fusifor, 19.6 Right-Cerebellum-Cortex, 9.4 ctx_rh_S_oc-temp_med_and_Lingual, 43.4 ctx-rh-fusiform, 30.0 Right-Cerebral-White-Matter, 17.1 Right-Cerebellum-Cortex, 6.5 ctx-rh-lingual,

12 297 vox [24 19 45]: 43.7 Right Superior Frontal Gyrus, 34.7 Right Middle Frontal Gyrus, 25.2 Right Brodmann area 8, 6.2 Right Brodmann area 6, 51.7 Right Superior Frontal Gyrus, 45.6 Right Middle Frontal Gyrus, 93.7 Right Brain, 39.9 Right-Cerebral-White-Matter, 37.7 ctx_rh_S_front_sup, 15.8 ctx_rh_G_front_middle, 6.1 ctx_rh_G_front_sup, 53.1 Right-Cerebral-White-Matter, 29.4 ctx-rh-superiorfrontal, 13.7 ctx-rh-caudalmiddlefrontal,

13 291 vox [26 -67 -33]: 30.5 Right Cerebellar Tonsil, 23.1 Right Pyramis, 12.9 Right Uvula, 12.9 Right Tuber, 8.8 Right Inferior Semi-Lunar Lobule, 59.0 Right Cerebellum (Crus 1), 28.9 Right Cerebellum (Crus 2), 74.5 Lobule VIIa Crus I (Hem), 12.3 Lobule VIIa Crus II (Hem), 91.7 Right Brain, 89.4 Right-Cerebellum-Cortex, 89.4 Right-Cerebellum-Cortex,

14 275 vox [-56 -32 38]: 72.2 Left Inferior Parietal Lobule, 13.7 Left Postcentral Gyrus, 44.3 Left Brodmann area 40, 8.0 Left Brodmann area 2, 33.5 Left SupraMarginal Gyrus, 23.4 Left Inferior Parietal Lobule, 38.3 IPC (PF), 15.8 IPC (PFt), 58.8 Left Brain, 70.7 ctx_lh_G_pariet_inf-Supramar, 17.1 Left-Cerebral-White-Matter, 78.4 ctx-lh-supramarginal, 10.4 Left-Cerebral-White-Matter,

15 236 vox [1 -85 -10]: 46.0 Right Lingual Gyrus, 27.8 Left Lingual Gyrus, 23.2 Right Brodmann area 18, 15.5 Left Brodmann area 18, 47.2 Left Calcarine Gyrus, 23.4 Right Lingual Gyrus, 12.2 Left Lingual Gyrus, 48.9 Area 17, 32.9 Area 18, 57.1 Left Brain, 37.9 Right Brain, 38.3 Left-Cerebral-White-Matter, 24.8 Right-Cerebral-White-Matter, 20.9 ctx_rh_Pole_occipital, 6.2 ctx_lh_Pole_occipital, 42.0 Left-Cerebral-White-Matter, 33.4 Right-Cerebral-White-Matter, 12.5 ctx-rh-lingual, 5.9 ctx-lh-lateraloccipital,

16 211 vox [9 -58 -42]: 24.2 Right Cerebellar Tonsil, 13.9 Left Cerebellar Tonsil, 6.7 Right Inferior Semi-Lunar Lobule, 21.2 Cerebellar Vermis (8), 19.5 Right Cerebellum (IX), 17.4 Right Cerebellum (VIII), 9.6 Cerebellar Vermis (9), 5.1 Right Cerebellum (VII), 35.2 Lobule IX (Hem), 16.2 Lobule VIIIb (Vermis), 13.2 Lobule VIIIa (Vermis), 11.7 Lobule IX (Vermis), 7.6 Lobule VIIb (Hem), 79.1 Right Brain, 17.6 Left Brain, 76.4 Right-Cerebellum-Cortex, 15.6 Left-Cerebellum-Cortex, 76.4 Right-Cerebellum-Cortex, 15.6 Left-Cerebellum-Cortex,

## S2.2 Associations of regions with behavioral data

To examine whether these regions were associated with behavioral ratings, mean savoring and rumination were correlated with neural reactivity during savoring and rumination respectively. At the voxelwise p<.005, 198 voxel cluster threshold level, there were no significant clusters of association with savoring. At the exploratory 100 voxel cluster threshold level, a mid-cingulate region was associated with savoring (**Figure S2.1;** 164 voxels centroid -4, 6.5, 50.5**)**; this region did not intersect with the savoring map (and thus also did not overlap with the the conjunctive map), similarly thresholded**.** For rumination related reactivity, no clusters larger than 30 voxels were associated with mean rumination ratings.

**
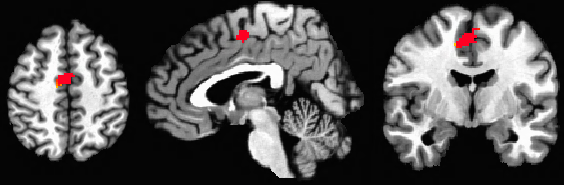
**

Figure S2.1. Regions during savoring associated with mean savoring behavioral response

## S2.3 Subthreshold regions for Savoring vs. Rumination

To account for the potential that differences between savoring and rumination have a weaker signal than was detected using our analytical framework, we also include sensitivity analyses showing differences between the conditions which emerge when the threshold for significance or contiguity threshold is decreased below that necessary for brain-wise p<.05, controlling for type I error across the brain. The emergent regions can be used in replication studies which define specific a priori regions and are available from the authors upon request.

As shown in Figure S2.2, when the contiguity threshold was relaxed from the empirically determined 198 voxels, other regions were revealed. At over 45 voxels, regions which were significantly greater for savoring than rumination included the previously determined motor regions (paracentral lobule and left postcentral gyrus) as well as a mid-cingulate region (cenroid -4, -10.5, 48.5; 116 voxels), a cerebellar region (14, -68.6, -49.5; 92 voxels), lingual gyrus (-20, 72.5, -3.5, 69 voxels), right anterior insula (36, 21.5, 2.5; 66 voxels), other motor areas, superior frontal gyrus (-22, 13.5, 52.5; 52 voxels), right posterior insula (40, -32.5, 20.5; 48 voxels), and the precuneus (12, 76.5, 12.5). No such regions were greater for rumination than savoring. Using a 45 voxel threshold revealed a middle temporal region (-48, -8.5, -19.5; 45 voxels) and cerebellar region (-44, -44, -51.5; 45 voxels) which were more strongly activated for rumination than savoring. These same regions were largely present when other methods for relaxing type I error control (e.g., p<.01, 100 voxels contiguity) were employed.

As shown in Figure S2.3, relaxing the statistical threshold further to 0.5*median of the upper 65% of values, which is often a reasonable heuristic for the types of effects that could turn up upon strong replication, the executive network (DLPFC, cingulate, parietal regions) as well as some salience network (amygdala, insula) regions were more active for savoring. Rumination was primarily more active throughout the lower part of the cerebellum.

**Figure S2.2**. Savoring vs. rumination, p<.005, 20 voxels contiguity

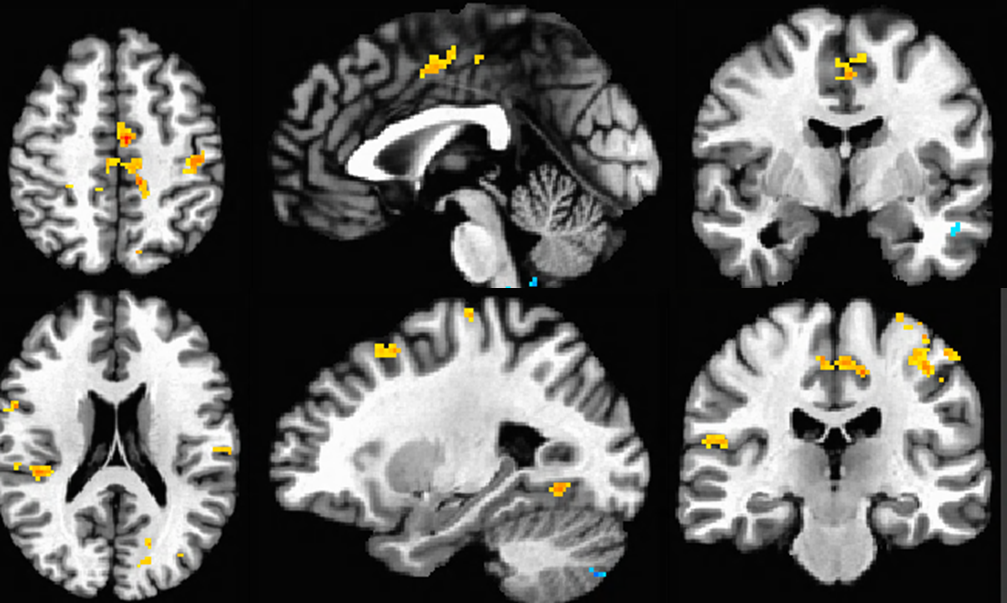


Figure S2.3: Savoring vs. Rumination using AFNI’s autothresholding at 0.5*median of the upper 65% of positive values, 1000 voxels contiguity thresholding

**
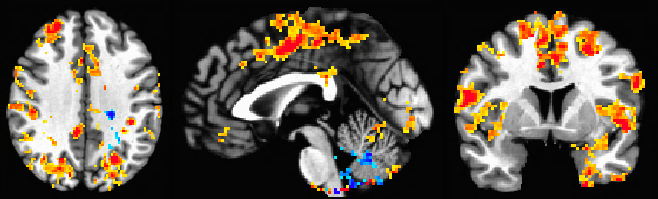
**

Thus, it is possible that a well-powered study directed, specifically, at detecting differences between savoring and rumination would find these networks.

# Supplement 3: Sub-cluster-threshold regions associated with specific memories.

Figures are colored based on R^2 from simultaneous regressions of specific memories for savoring and rumination, for each of the conditions.

**Savoring: p<.05, 100 voxels contiguity threshold (theoretically determined)**

**
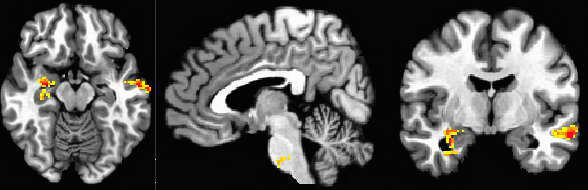
**

**Savoring, p<.005, 10 voxels contiguity threshold (theoretically determined)

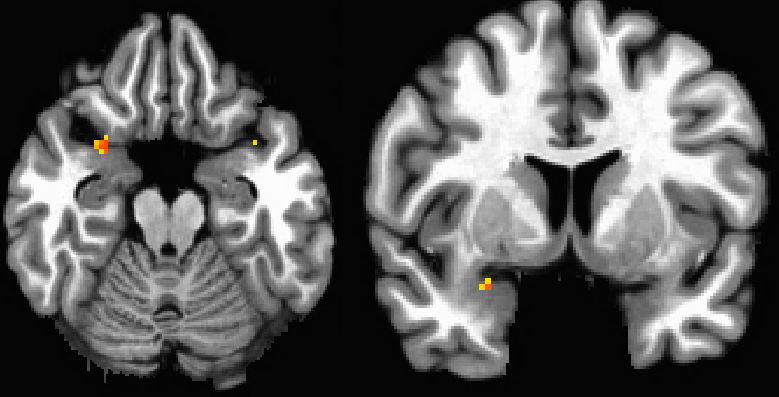
**

**Rumination: p<.05, 100 voxels contiguity threshold (theoretically determined)**

**
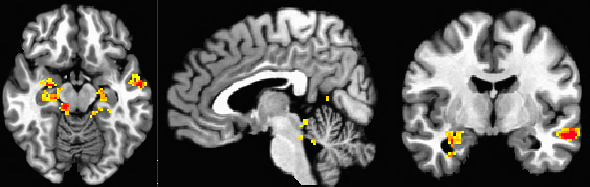
**

**Rumination, p<.01, 5 voxels contiguity threshold (theoretically determined)**

**
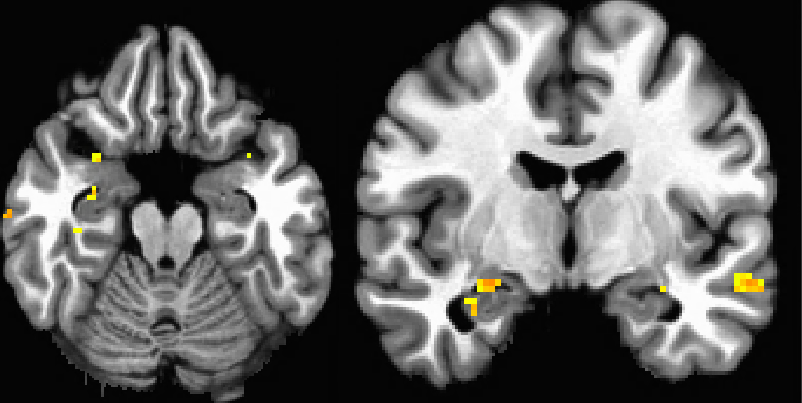
**

# Supplement 4: Sub-threshold associations with Sleep

Voxelwise analyses of associations with PROMIS sleep disturbance with reactivity during each of rumination and savoring found no regions that exceeded cluster thresholding at p<.005 to yield effective p<.05. Results thresholded at p<.005 and 20 voxels contiguity are shown to avoid file drawer issues in case these areas are replicated in other studies.


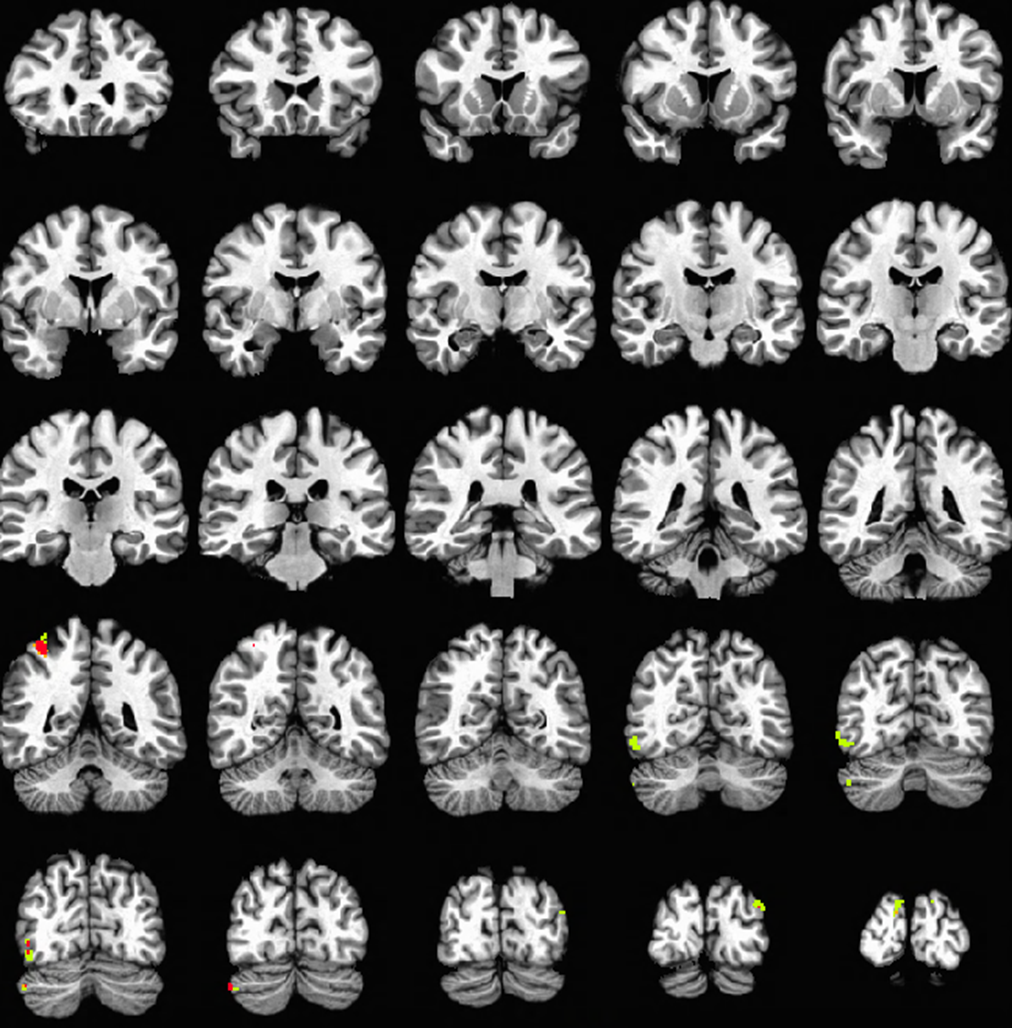


Figure S4.1: Regions in which sleep disturbance was associated with savoring (green), ruminating (orange) or both savoring and rumination (red), p<.005, 20 voxels contiguity

**Table S4.1.** Regionwise centroids for savoring-sleep, and rumination-sleep, and their conjunction correlation maps, p<.005, 20 voxels contiguity thresholded

**#Voxels CM x CM y CM z Peak x Peak y Peak z**

**Savor**

93 -51.9 +68.9 -11.4 -54.0 +66.5 -15.5

62 -36.4 +49.5 +61.6 -34.0 +48.5 +68.5

47 -49.6 +73.5 -37.6 -50.0 +78.5 -33.5

36 +9.7 +99.0 +20.9 +6.0 +102.5 +18.5

28 -12.6 +94.3 +24.0 -10.0 +94.5 +26.5

28 +37.3 +88.6 +22.3 +36.0 +90.5 +24.5

22 -12.4 +102.7 +4.6 -10.0 +102.5 +2.5

21 +59.5 +25.4 -6.8 +60.0 +24.5 -7.5

**Ruminate**

55 -36.9 +49.2 +59.9 -38.0 +50.5 +64.5

22 -11.0 +90.5 +29.1 -8.0 +88.5 +34.5

**Savor and Ruminate**

44 -36.6 +49.7 +60.2 -34.0 +48.5 +54.5

#

# Supplement 5: Instructions for generating scripts for the Savoring and Rumination task

**Rumination**

In the next part of the session we are interested in how the brain processes different types of thinking.

We will ask you to ruminate on negative things about you three times. To help with this, we would like you to write down three short descriptions to think about. These descriptions might regard characteristics or traits of yours that make you unhappy, negative themes that comes up over and over in your life, or situations for which you feel bad about your role.

They should represent the types of things you think about when you are upset, down or depressed. On a scale of 1 (neutral) to 9 (extremely sad/upset) we ask that you try to pick thoughts you would rate as at least a 7. That is, it is important that these are descriptions that make you ruminate in a sad/upset way.

Examples might be something you feel very guilty about, something you do not like about yourself, or something negative that other people say about you.

We will ask you to read these scripts in the scanner, and to allow yourself to ruminate on them. Please try to pick thoughts that are strong enough to make you ruminate, and to describe them in a way that you will be able to ruminate upon in the scanner.

Please note: They will NOT be used or shared with other participants.

1. _____________________________________________________________________

_____________________________________________________________________

_____________________________________________________________________

How **upset, down, depressed, or sad** are you when you think about this topic? (circle the one best number):

1 ………. 2 ………. 3 ………. 4 ………. 5 ………. 6 ………. 7 ………. 8 ………. 9

Neutral Slightly Moderately Very Extremely

1. _____________________________________________________________________

_____________________________________________________________________

_____________________________________________________________________

How **upset, down, depressed, or sad** are you when you think about this topic? (circle the one best number):

1 ………. 2 ………. 3 ………. 4 ………. 5 ………. 6 ………. 7 ………. 8 ………. 9

Neutral Slightly Moderately Very Extremely

1. _____________________________________________________________________

_____________________________________________________________________

_____________________________________________________________________

How **upset, down, depressed, or sad** are you when you think about this topic? (circle the one best number):

1 ………. 2 ………. 3 ………. 4 ………. 5 ………. 6 ………. 7 ………. 8 ………. 9

Neutral Slightly Moderately Very Extremely

**Positive Reflection**

In the next part of the session we are interested in how the brain processes different types of thinking.

We will ask you to reflect on positive things about you three times. To help with this, we would like you to write down three short descriptions to think about. These descriptions might regard characteristics or traits of yours that make you happy, positive themes that come up over and over in your life, or situations for which you feel happy about your role.

They should represent the types of things you think about when you are happy or in a good or positive mood. On a scale of 1 (neutral) to 9 (extremely happy/positive) we ask that you try to pick thoughts you would rate as at least a 7. That is, it is important that these are descriptions that make you reflect in a happy/positive way.

Examples might be something you feel very happy about, something you like about yourself, or something positive that other people say about you.

We will ask you to read these scripts in the scanner, and to allow yourself to reflect on them. Please try to pick thoughts that are strong enough to make you reflect, and to describe them in a way that you will be able to reflect upon in the scanner.

Please note: They will NOT be used or shared with other participants.

1. _____________________________________________________________________

_____________________________________________________________________

_____________________________________________________________________

How **happy, positive, or uplifted** are you when you think about this topic? (circle the one best number):

1 ………. 2 ………. 3 ………. 4 ………. 5 ………. 6 ………. 7 ………. 8 ………. 9

Neutral Slightly Moderately Very Extremely

1. _____________________________________________________________________

_____________________________________________________________________

_____________________________________________________________________

How **happy, positive, or uplifted** are you when you think about this topic? (circle the one best number):

1 ………. 2 ………. 3 ………. 4 ………. 5 ………. 6 ………. 7 ………. 8 ………. 9

Neutral Slightly Moderately Very Extremely

1. _____________________________________________________________________

_____________________________________________________________________

_____________________________________________________________________

How **happy, positive, or uplifted** are you when you think about this topic? (circle the one best number):

1 ………. 2 ………. 3 ………. 4 ………. 5 ………. 6 ………. 7 ………. 8 ………. 9

Neutral Slightly Moderately Very Extremely

# Supplement 6: Details of Memory Specificity Coding

A specific memory was defined as memory for a single event that took place at an identified place and did not last longer than 1 day (e.g. ‘attending Jane’s party’). A categorical memory was defined as a memory referring to a category of events containing a number of specific episodes, without reference to a single event (e.g. ‘ all the times I’ve failed an exam’ without reference to a specific occurrence where a test was failed). An extended memory was defined as a memory for an extended period of time (e.g. a semester at school). A semantic memory was defined as a fact (examples include statements without associated events, such as ‘ I have never been to a dance’). A repeat memory was defined as a memory a participant used for a previous cue word. Finally, no memory was used when a participant could not recall a memory at all. All responses were rated by one rater.

# Supplement 7: Association of individual differences with affect intensity ratings

We examined whether there were likely sources of individual differences which accounted for variation in affect intensity ratings across individuals using eight candidate trait self-report measures as well as sad affect reported during the scan prior to and after the task.

## S7.1 Methods

The trait self-reported measures used were depression symptomatology, emotional distress, trait rumination, worry, state anxiety, dampening of positive affect, emotional responses to positive affect, and self focus in response to positive affect.

**Depression symptomatology.** The Inventory of Depressive Symptomatology Self-Report (IDSR) [(Rush et al. 1986, 1996)](https://paperpile.com/c/2EHe8F/78ZYv+QzYRB) is a self-report measure of depression severity with 30 items scored from 0 to 3 and summed to yield a total score between 0 and 84 with a higher score indicating more severe depressive symptomatology. The total score correlates with other known measures of depression such as the Beck Depression Inventory.

**Emotional distress.** The PROMIS emotional distress scale score [(Pilkonis et al. 2011; Kiken and Shook 2012; Cella et al. 2007)](https://paperpile.com/c/2EHe8F/xvSu+StzW+2LBZ)**)** contains 15 total items assessing depression and anxiety with each item scored from 1 to 5 summed to yield a total score ranging from 15 to 75 with a higher score indicating more emotional distress**.**

**Trait rumination.** The Response Styles Questionnaire, Rumination Scale [(Nolen-Hoeksema and Morrow 1991)](https://paperpile.com/c/2EHe8F/zsBd4) is a measure of trait rumination in which rumination is operationally defined as thinking about one’s negative emotions. It contains 22 items, each scored from 1 to 4 summing to yield a total score between 22 and 88 with a higher score indicating more ruminatory symptoms.

**Worry.** The Penn State Worry Questionnaire [(Meyer et al. 1990)](https://paperpile.com/c/2EHe8F/G9GSo) was designed to assess worry. It contains 16 items which are scored from 1 to 5 summed to yield a total score ranging from 16 and 80 with a higher score indicating more worry.

**State anxiety.** The Spielberger State Anxiety Inventory [(Speilberger et al. 1983)](https://paperpile.com/c/2EHe8F/0QX8A) has scales to assess the severity of a person’s state anxiety. It contains 40 items which are scored from 0 to 3 and are summed to yield a total score ranging from 0 to 120 with a higher score indicating higher levels of anxiety.

**Responses to positive affect.** The Responses to Positive Affect Scale [(Feldman, Joormann, and Johnson 2008)](https://paperpile.com/c/2EHe8F/lQsHc) assesses factors related to how people respond to positive affect, operationally defined as thinking about one’s positive emotions including emotional responses such as rumination as well as self-focus. Items are scored from 1 to 4 and summed within scales to yield scale scores. There are 7 items pertaining to **dampening** (or consciously diminishing affective intensity; scores range from 7-28), 5 items for **emotion focus** (akin to emotion-focused rumination, for positive information; scores range from 5-20)**,** and 4 items for **self-focus**  (scores range from 4-16).

**Sad affect** prior to and after the task were rated using a five point likert scale (not at all sad to very sad) entered using the same mouse used for the task.

## S7.2 Results

Table S7.1 shows these correlations with mean affect during rumination and savoring. No correlations in the table below were statistically significant (all p’s>.05).

|  | Correlation (r) with mean affect rating during Rumination | Correlation (r) with mean affect rating during Savoring |
| --- | --- | --- |
| Depressive symptomatology (IDSR) | -0.11 | -0.03 |
| PROMIS Emotional Distress | .05 | -.24 |
| Trait Rumination (as measured by the Response Styles Questionnaire, Rumination Scale) | -.18 | .02 |
| Worry (Penn State Worry Questionnaire) | -.12 | -.14 |
| State Anxiety (Spielberger State Anxiety) | -.15 | -.14 |
| Dampening of positive affect (Responses to Positive Affect Scale) | -.19 | .04 |
| Emotional responses to positive affect (Responses to Positive Affect Scale) | .11 | .03 |
| Self-focus in response to positive affect (Responses to Positive Affect Scale) | .03 | .20 |
| Sad affect reported prior to the task | -.19 | -.21 |
| Sad affect reported after the task | -.04 | -.10 |

# Supplement 8: Associations of subjective rating means and range-measures

It is possible that mean scores on savoring and rumination are related by a response style by which people who use more of the full range on one scale are also prone to use more of the full range on any other scale.

To see how strong the evidence was for this type of theory, we examined associations of the mean ratings on savoring and rumination with various ways of quantifying use of the range of ratings (Table S.8.1). The table shows that the means of savoring and rumination were not correlated with the standard deviation, within condition, for either savoring or rumination, which argues against this explanation. That said, the means of savoring and rumination were associated with the standard deviation and range of the data, which is expected given that moods started at neutral at the beginning of blocks and thus covered the range from their starting point to their mean.

Table S.8.1 shows correlations of the mean of subjective ratings of each condition, the standard deviation of each condition, and the standard deviation of the entire rating waveform along with its 5th and 95th percentiles and the range between the 5th and 95th percentile.


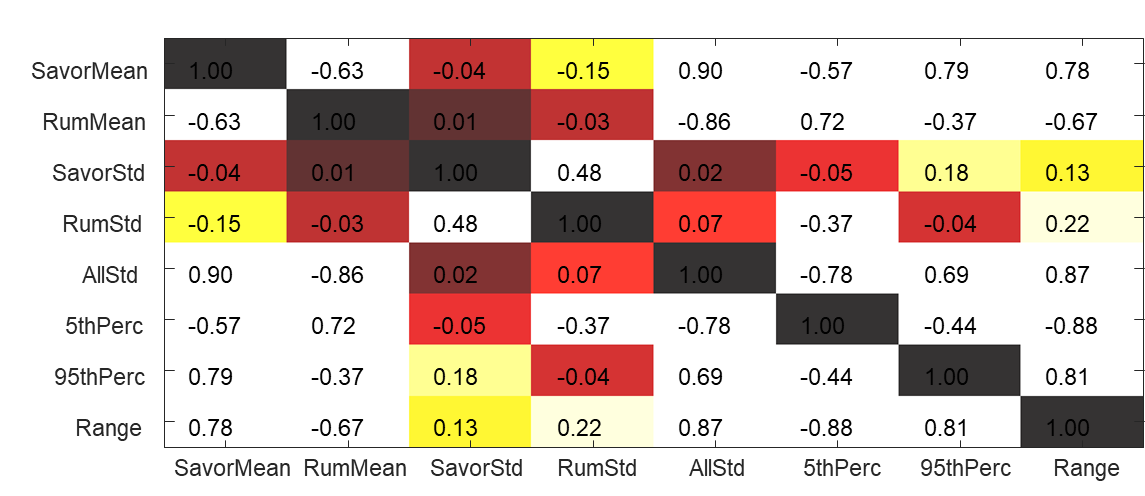


To show that this question is inherently hard to answer with the current data because higher means for savoring and lower means for rumination will inherently yield larger use of the range, we examined associations of the means of simulated random walks with the standard deviation and range measures on those random walks, calculated exactly as we did in Table S.8.1 for N=60 simulated participants. As shown in Table S.8.2, correlation of the mean and the range measures were on par between the simulated and real data, suggesting that it is hard to interpret these types of correlations as reflecting a true bias.

Table S.8.2 shows analogous correlations to those shown in Table 1, for simulated random walk data, showing that high levels of association are expected between the means and ranges of randomly generated autocorrelated rating data.


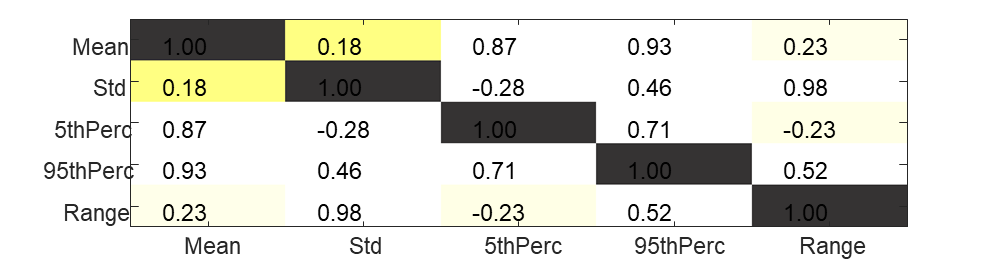


Thus, future research using an experimental design more strongly tuned to this question is necessary to understand the extent to which a bias towards wider-range responding would explain the obtained pattern of results.

# References for Supplement

[Cella, David, Susan Yount, Nan Rothrock, Richard Gershon, Karon Cook, Bryce Reeve, Deborah Ader, et al. 2007. “The Patient-Reported Outcomes Measurement Information System (PROMIS): Progress of an NIH Roadmap Cooperative Group during Its First Two Years.” *Medical Care* 45 (5 Suppl 1): S3–11.](http://paperpile.com/b/2EHe8F/2LBZ)

[Feldman, Greg C., Jutta Joormann, and Sheri L. Johnson. 2008. “Responses to Positive Affect: A Self-Report Measure of Rumination and Dampening.” *Cognitive Therapy and Research* 32 (4): 507–25.](http://paperpile.com/b/2EHe8F/lQsHc)

[Kiken, Laura G., and Natalie J. Shook. 2012. “Mindfulness and Emotional Distress: The Role of Negatively Biased Cognition.” *Personality and Individual Differences* 52 (3): 329–33.](http://paperpile.com/b/2EHe8F/StzW)

[Meyer, T. J., M. L. Miller, R. L. Metzger, and T. D. Borkovec. 1990. “Development and Validation of the Penn State Worry Questionnaire.” *Behaviour Research and Therapy* 28 (6): 487–95.](http://paperpile.com/b/2EHe8F/G9GSo)

[Nolen-Hoeksema, Susan, and Jannay Morrow. 1991. “A Prospective Study of Depression and Posttraumatic Stress Symptoms after a Natural Disaster: The 1989 Loma Prieta Earthquake.” *Journal of Personality and Social Psychology* 61 (1): 115–21.](http://paperpile.com/b/2EHe8F/zsBd4)

[Pilkonis, Paul A., Seung W. Choi, Steven P. Reise, Angela M. Stover, William T. Riley, David Cella, and PROMIS Cooperative Group. 2011. “Item Banks for Measuring Emotional Distress from the Patient-Reported Outcomes Measurement Information System (PROMIS®): Depression, Anxiety, and Anger.” *Assessment* 18 (3): 263–83.](http://paperpile.com/b/2EHe8F/xvSu)

[Rush, A. J., D. E. Giles, M. A. Schlesser, C. L. Fulton, J. Weissenburger, and C. Burns. 1986. “The Inventory for Depressive Symptomatology (IDS): Preliminary Findings.” *Psychiatry Research* 18 (1): 65–87.](http://paperpile.com/b/2EHe8F/78ZYv)

[Rush, A. J., C. M. Gullion, M. R. Basco, R. B. Jarrett, and M. H. Trivedi. 1996. “The Inventory of Depressive Symptomatology (IDS): Psychometric Properties.” *Psychological Medicine* 26 (3): 477–86.](http://paperpile.com/b/2EHe8F/QzYRB)

[Speilberger, C. D., R. L. Gorsuch, R. Lushene, and P. R. Vagg. 1983. “Manual for the State-Trait Anxiety Inventory.” *Palo Alto, CA: Consulting*.](http://paperpile.com/b/2EHe8F/0QX8A)
